# Repeated Nipple Fluid Aspiration: Compliance and Feasibility Results from a Prospective Multicenter Study

**DOI:** 10.1371/journal.pone.0127895

**Published:** 2015-05-22

**Authors:** J. S. de Groot, C. B. Moelans, S. G. Elias, A. Hennink, B. Verolme, K. P. M. Suijkerbuijk, A. Jager, C. Seynaeve, P. Bos, A. J. Witkamp, M. G. E. M. Ausems, P. J. van Diest, E. van der Wall

**Affiliations:** 1 Department of Pathology, University Medical Center Utrecht, Utrecht, The Netherlands; 2 Julius Center for Health Sciences and Primary Care, University Medical Center Utrecht, Utrecht, The Netherlands; 3 Department of Medical Oncology, Erasmus University Medical Center, Daniel den Hoed Cancer Center, Rotterdam, The Netherlands; 4 Department of Surgery, University Medical Center Utrecht, Utrecht, The Netherlands; 5 Department of Medical Genetics, University Medical Center Utrecht, Utrecht, The Netherlands; 6 Department of Medical Oncology, University Medical Center Utrecht, Utrecht, The Netherlands; The Institute of Cancer Research, UNITED KINGDOM

## Abstract

**Background:**

Despite intensive surveillance, a high rate of interval malignancies is still seen in women at increased breast cancer risk. Therefore, novel screening modalities aiming at early detection remain needed. The intraductal approach offers the possibility to directly sample fluid containing cells, DNA and proteins from the mammary ductal system where, in the majority of cases, breast cancer originates. Fluid from the breast can non-invasively be obtained by oxytocin-assisted vacuum aspiration, called nipple fluid aspiration (NFA). The goal of this feasibility study was to evaluate the potential of repeated NFA, which is a critical and essential step to evaluate its possible value as a breast cancer screening method.

**Methods:**

In this multicenter, prospective study, we annually collected nipple fluid for up to 5 consecutive years from women at increased breast cancer risk, and performed a questionnaire-based survey regarding discomfort of the aspiration. Endpoints of the current interim analyses were the feasibility and results of 994 NFA procedures in 451 women with total follow-up of 560 person years of observation.

**Results:**

In this large group of women at increased risk of breast cancer, repetitive NFA appeared to be feasible and safe. In 66.4% of aspirated breasts, nipple fluid was successfully obtained. Independent predictive factors for successful NFA were premenopausal status, spontaneous nipple discharge, smaller breast size, bilateral oophorectomy and previous use of hormone replacement therapy or anti-hormonal treatment. The procedure was well tolerated with low discomfort. Drop-out rate was 20%, which was mainly due to repeated unsuccessful aspiration attempts. Only 1.6% of women prematurely declined further participation because of side effects.

**Conclusions:**

Repeated NFA in women at increased breast cancer risk is feasible and safe. Therefore, NFA is a promising method to non-invasively obtain a valuable source of potential breast cancer specific biomarkers.

## Introduction

Breast cancer causes the highest cancer related mortality in women with 458,000 deaths worldwide in 2008 [1]. Moreover, the incidence of breast cancer is high with a lifetime risk of approximately 13% for a woman in The Netherlands. This risk increases dramatically in women carrying a breast cancer susceptibility gene, such as *TP53*, *PTEN*, *LKB1*, *CDH1*, or, most frequently, *BRCA1* or *BRCA2* [2]. Women carrying a germline *BRCA1* mutation have a 57–65% chance of developing breast cancer before the age of 70 years, which for *BRCA2* mutation carriers is 45–55% [3–5]. After having developed unilateral breast cancer, *BRCA1/2* mutation carriers are also at increased risk of subsequent contralateral breast cancer. This risk is estimated to be 20–60% or even higher and is influenced by various factors such as age at diagnosis and adjuvant systemic therapy [5,6].

To date, the most effective preventive options in women at increased breast cancer risk are bilateral or contralateral prophylactic mastectomy (PM) and/or prophylactic bilateral salpingo-oophorectomy (PBSO). PM yields a risk reducing effect of more than 95% in healthy *BRCA1/2* mutation carriers [7]. PBSO before the age of 50 years decreases breast cancer risk in *BRCA1/2* mutation carriers without prior breast cancer (HR 0.36–0.63) [8]. Data about the breast cancer reducing effect of PBSO in postmenopausal women are conflicting [8,9]. An alternative preventive strategy could be chemoprevention with anti-hormonal therapy, but evidence of efficacy so far is marginal [10].

Another option to prevent breast cancer related mortality in high risk women is intensive surveillance including e.g. mammography and MRI. In follow-up studies, annual MRI significantly reduced the incidence of advanced-stage breast cancers in *BRCA1/*2 mutation carriers [11] and detected the majority of breast cancers at an early and favorable stage [12]. MRI is more sensitive but less specific than mammography in detecting invasive breast cancer. Strikingly, the sensitivity of mammography in diagnosing breast cancer is lower in *BRCA1* mutation carriers compared to *BRCA2* mutation carriers or women with a moderate to high familial breast cancer risk [13]. Moreover, *BRCA1* mutation carriers have more interval cancers (32%) and an unfavourable tumor size at diagnosis [13]. Other disadvantages of breast cancer screening are difficulties in interpreting imaging in women with dense breasts, false positive results leading to additional examinations and higher costs, and more distress [14–16].

The intraductal approach offers a way to directly access or sample fluid from the mammary ductal system, where in the majority of patients breast cancer develops. Nipple fluid contains cells, DNA and proteins directly derived from the breast ducts and can thereby be a rich source of breast cancer biomarkers [17,18]. Fluid from the breast can be obtained by invasive techniques like random fine needle aspiration (FNA) or ductal lavage (DL), but nipple fluid can also be obtained in a completely non-invasive way by an oxytocin-assisted nipple fluid aspiration (NFA) under vacuum. Besides being less invasive, NFA causes less discomfort and is easier to perform compared to invasive techniques [19]. We have previously shown that, with this technique, nipple fluid can be obtained successfully and without discomfort in healthy women and women at increased risk of breast cancer [20,21].

In the present analyses, we investigated the feasibility of and variables affecting a successful NFA procedure in a prospective, multicenter study where nipple fluid was obtained annually in women at increased breast cancer risk adhering to a surveillance program. Also, compliance and discomfort associated with the procedure was studied. The goal of this clinical feasibility study was to evaluate the potential of repeated nipple fluid aspiration, which is a critical and essential step to evaluate its possibility as a breast cancer screening method.

## Methods

### Study protocol and population

Women at increased risk of breast cancer adhering to a regular surveillance program were included in this prospective clinical study aiming to establish methylation profiles in nipple fluid. According to the Dutch guidelines, the surveillance program provides imaging based screening as standard of care for women at increased breast cancer risk based on inheritance or a history of breast cancer. The study design is observational: a cohort of high-risk women is being followed from baseline to the end of follow-up or until development of breast cancer or (preventive) breast surgery. Nipple fluid was aspirated annually, for a follow-up period of five years. Besides ending the follow-up period of five years, participation could be discontinued because of the development of breast cancer, breast surgery making NFA impossible, or the exclusion of a *BRCA1/2* mutation after genetic testing. Person years of observation were calculated from baseline to the last NFA procedure or last moment of contact with the participant.

Apart from the NFA, participants had their regular follow-up consultations with the physician or nurse practitioner and their imaging examinations. The indication for breast surgery was based on usual clinical and radiological findings, and some patients opted for risk reducing mastectomy at which time the follow-up into the study stopped. Physicians and patients remained blinded to the results of the nipple aspirate analyses. Enrolment of participants started at August 7, 2008 in the University Medical Center Utrecht (UMCU) and at April 22, 2011 in the Erasmus Medical Center Rotterdam (EMC).

The study has been approved by the Ethics Committees of the UMCU and the EMC, The Netherlands (ABR NL 11690.041.06, METC 06–091). Written informed consent was obtained from all participants.

The objective of the present analysis was to assess the feasibility and compliance of repeated NFA, the variables predictive for a successful aspiration of nipple fluid, and discomfort experienced by participants in comparison with other surveillance procedures and breast feeding.

### Study population

Women at increased risk of developing (a new) breast cancer adhering to a surveillance program were eligible for the study, including: carriers of a *BRCA1* or *BRCA2* gene mutation, women with a pedigree-based increased lifetime breast cancer risk, or a history of DCIS or invasive breast cancer. Exclusion criteria were age below 18 years, bilateral mastectomy, pregnancy or lactation, active breast infection, and disseminated breast cancer.

### Nipple fluid aspiration technique

**Fig 1:**
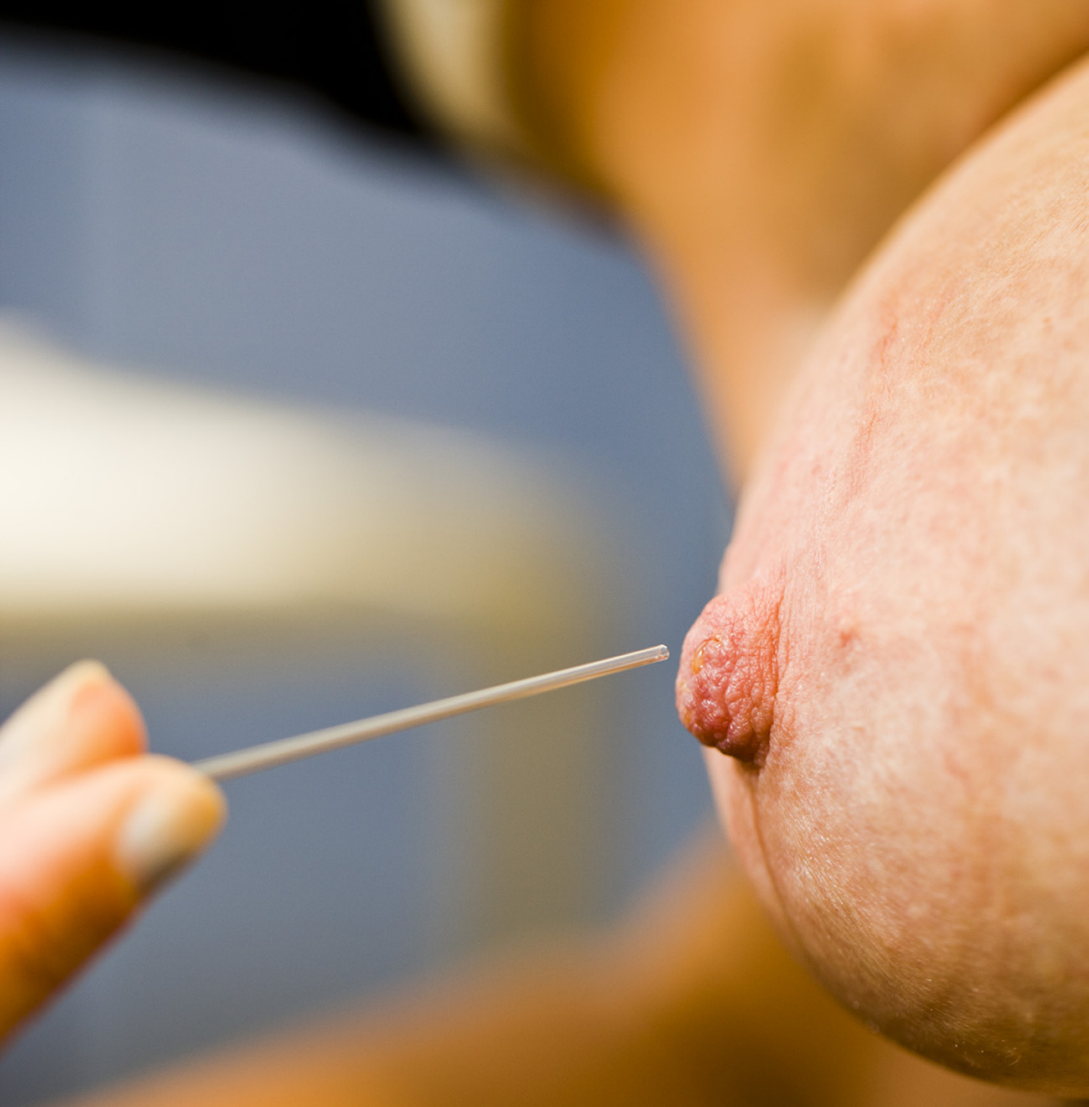
Nipple fluid collected by a capillary tube after nipple fluid aspiration (NFA).

The technique of NFA has been previously described [17,21]. In short, anaesthetic cream (Emla) was applied onto the nipple, after which the breasts were warmed with hot pads. Women were then administered oxytocin nasal spray into both nostrils (see below). A suction cup (aspirator; one-day pump set manufactured by Medela; as from December 2012 one-day pump set manufactured by Beldico because of supply issues) was placed over the nipple. Repeated, intermittent manual gentle suction with a 20 cc syringe connected to the suction cup drew fluid to the nipple surface. If necessary, suction was applied for 20 to 30 minutes. Droplets were collected by capillary tubes (Fig 1). The entire procedure was applied to each breast separately. The collected fluid from different ducts of each breast was pooled and conserved in a buffer solution (50mM Tris pH 8.0, 150mM NaCl, 2mM EDTA) at -80°C until analysis. The procedure was called successful if droplets were visible on the surface of the nipple and could be collected with the capillary tube.

To test the feasibility of obtaining nipple fluid with an electric pump, we also performed a pilot study in 43 women in which vacuum was applied simultaneously to both breasts using an electric device (Medela Symphony, Baar, Switzerland) for approximately 15–20 minutes. If after 10 minutes no droplets were seen, a second dose of oxytocin was given and the aspiration procedure was repeated.

### Oxytocin hormonal nasal spray

Participants received oxytocin nasal spray (Syntocinon) in a dose of 4 IU per spray in order to stimulate the production of nipple fluid. One spray of nasal oxytocin contains 4 IU and is the standard dose to induce lactation in breastfeeding women. In the mammary gland, oxytocin induces contraction of the myoepithelial cells which surround the milk-storing alveoli and in this way facilitates the release of milk from the breast during lactation [22]. Moreover, oxytocin causes rhythmic contractions of the uterus during labour and has been shown to play a role in the central nervous system regulating for example maternal, sexual and social behaviour [22].

Reported adverse events in the Summary of Product Characteristics of nasal oxytocin include headache (<1/1,000), nausea (<1/1,000), allergic dermatitis (<1/1,000), and uterine contractions (<1/100). Syntocinon is quickly absorbed by the nasal mucosa and effective 5 minutes after administration. In case of supratherapeutic dosing, the oxytocin will be swallowed and degraded quickly by proteolytic enzymes in the gastro-intestinal tract. Oxytocin has a half-life that varies from 3 to 20 minutes and it is mainly eliminated by the liver and the kidneys [23].

### Questionnaires

A questionnaire addressing age, phase in menstrual cycle, menarche, menopause, use of oral anticonceptives or hormonal replacement therapy, parity, breast feeding, spontaneous nipple discharge, prior mammography, prior palpable masses in the breast, biopsy or breast surgery, oophorectomy and chemo- or radiotherapy, was filled out before the first NFA procedure, and updated at every subsequent visit. Spontaneous nipple discharge is defined as physiologic discharge which is spontaneous, usually bilateral, involves multiple ducts and does not contain blood. Around 50%-80% of women in their reproductive years can express fluid from their breasts. Spontaneous nipple discharge is different from pathological nipple discharge, which is unilateral, and can be bloody, serous, clear, or associated with a mass [24]. A discomfort questionnaire was completed by the participant together with the research nurse after every NFA procedure where discomfort was scored on a scale from zero (no discomfort) to ten (worst imaginable discomfort). The discomfort questionnaire was based on the pain questionnaire for the evaluation of NFA developed by Klein *et al*. [25]. In order to be able to put the experienced discomfort during NFA into perspective, discomfort of other breast examination procedures and breast feeding was asked and compared with the NFA procedure.

### Statistical analyses

For statistical analyses IBM SPSS Statistics Version 20 was used. A two-sided *P*-value <0.05 was considered statistically significant. To account for the clustered data, we analysed the nipple fluid aspiration procedure results using repeated measurement analysis by General Estimation Equations (GEE) with participant as the subject level and breast*visit as within-subject levels, using robust standard error estimation and accounting for within-subject dependencies assuming an autoregressive relationship. For categorical outcomes (aspect, volume, and number of drops of nipple fluid) we used a cumulative logit multinomial approach, and for success rate a negative binomial approach with a log link (providing accurate relative risk estimates for determinants in view of the high overall success rate). The reported overall frequency of the various outcomes and the frequency of success in patient subgroups are based on the GEE-estimated values. We used a stepwise-backward multivariable selection approach for determinants of aspiration success (through *P*<0.1). To compare electric and manual aspiration, we made use of paired analyses from the electric aspiration compared with a manual aspiration the visit before, or after if there was no earlier visit. Cases were excluded if only one visit with electronic aspiration was performed. To analyse discomfort rates, only the first visit was taken into account.

## Results

### Baseline characteristics of participants

At the time of analysis, a total of 994 NFA procedures had been performed in 451 women (UMCU: 292, EMC: 159). Since the Erasmus Medical Center started including participants from April 2011, only first and second year participants of this center were yet analysed. 295 women underwent a second NFA, 139 a third, and 84 a fourth. A total of 25 women completed the follow-up period of 5 years and 88% of them were willing to continue follow-up. Taken together there were 560 person years of observation at time of analysis.

Baseline characteristics and reasons for inclusion of participants are shown in Table 1. Mean age at inclusion was 47.9 years (median 48.0 years) with a range of 21 to 79 years.

**Table 1:** Baseline characteristics of 451 women at increased breast cancer risk undergoing (repeated) NFA.

Characteristic	Subgroups	*N*	%
Total		451	100
Age (years)	<40	107	23.7
	40–49	137	30.4
	≥50	207	45.9
Genetic status	No genetic examination performed	215	47.7
	No susceptibility factor detected	124	27.5
	*BRCA1* mutation	61	13.5
	*BRCA2* mutation	35	7.8
	Unclassified variant *BRCA*	1	0.2
	*BRCA1/2* in family	12	2.7
	*CDH1*	3	0.7
Lifetime breast cancer risk based on genetic status and family history^¶^	Standard*	125	27.7
	Moderate	60	13.3
	High	26	5.8
	Very high	113	25.1
	Unknown	127	28.2
Personal history of breast cancer	None	283	62.7
	DCIS	30	6.7
	Invasive carcinoma	138	30.6
History spontaneous nipple discharge	Yes	74	16.4
	No	377	83.6
Number of live births	0	115	25.5
	1–2	264	58.5
	≥3	72	16.0
Age at first birth (years)	<25	91	20.2
	25–29	132	29.3
	≥30	113	25.1
	Not applicable (nulliparous)	115	25.5
Previous breast feeding	Yes	268	59.4
	No	183	40.6
Age at menarche (years)	<12	138	30.6
	12–14	152	33.7
	≥14	156	34.6
	Unknown	5	1.1
Menopausal status	Premenopausal	209	46.3
	Postmenopausal	241	53.4
	Unknown	1	0.2
Age at menopause (years)	<45	82	18.2
	45–49	74	16.4
	≥50	85	18.8
	Not applicable (premenopausal)	209	46.3
	Unknown	1	0.2
Current oral contraceptive use	Yes	53	11.8
	No	398	88.2
History oral contraceptive use	Yes	416	92.2
	No	33	7.3
	Unknown	2	0.4
Current intrauterine device	Yes	20	4.4
	No	427	94.7
	Unknown	4	0.9
Current hormonal replacement therapy	Yes	10	2.2
	No	440	97.6
	Unknown	1	0.2
History hormonal replacement therapy	Yes	33	7.3
	No	414	91.8
	Unknown	4	0.9
Breast size	A-B	110	24.4
	C-D	283	62.7
	>D	53	11.8
	Unknown	5	1.1
Oophorectomy in history	Bilateral	95	21.1
	Unilateral	6	1.3
	No	350	77.6
Chemotherapy in history	Yes	88	19.5
	No	363	80.5
Radiotherapy in history	Yes	131	29.0
	No	320	71.0
Current anti-hormonal therapy	Yes	48	10.6
	No	397	88.0
	Unknown	6	1.3
History anti-hormonal therapy	Yes	62	13.7
	No	389	86.3
Breast surgery in history	Excision biopsy	30	6.6
	Breast conserving surgery	116	25.7
	Mastectomy	60	13.3
	Other	33	7.3

^¶^ Lifetime breast cancer risk was based on the Dutch guidelines for hereditary cancers as published by STOET (Stichting Opsporing Erfelijke Tumoren) and Vereniging Klinische Genetica Nederland in 2010 (http://stoet.nl/uploads/richtlijnenboekje.pdf)

* This group consists of women with a personal history of breast cancer

### Characteristics of obtained nipple fluid

The aspect of the obtained fluid was clear in 56.0%, cloudy in 31.0%, different colours from several ducts in 12.7%, and bloody in 0.3%. The estimated volume of the fluid aspirated was less than 5 μl in 37.1%, 5–50 μl in 59.6%, and more than 50 μl in 3.3% of women. In 74.8% of women 1 or 2 droplets were aspirated, in 21.2% 3 or 4 droplets, and in 4.0% 5 droplets or more.

### Success rates of nipple fluid aspiration

To analyse the influence of clinical characteristics on successful aspiration, analyses were done considering NFA per breast taking into account within-participant dependency between observations from each breast. NFA was performed in 1824 breasts and aspiration was successful in 66.4%. Table 2 shows the relation between baseline characteristics and success of NFA. Age, history of spontaneous nipple discharge, breast size, menopausal status, and current use of oral contraceptives were significantly correlated with success rate of NFA in univariate analysis. Using multivariate analysis postmenopausal status (RR = 0.74, CI95% 0.65–0.84), smaller breast size (RR = 1.11, CI95% 1.01–1.22), history of spontaneous nipple discharge (RR = 1.23, CI95% 1.11–1.36), bilateral oophorectomy (RR = 1.16, CI95% 1.01–1.34), and a history of hormone replacement therapy (RR = 1.27, CI95% 1.08–1.49) or anti-hormonal treatment (RR = 1.21, CI95% 1.04–1.42) independently predicted the success rate of NFA (Table 2).

**Table 2:** Predictive factors for successful NFA per 1824 attempts per breast using repeated measurement analysis.

Factor	Subgroup	Successful aspiration	*N*	Univariate analysis	Multivariate analysis
		%		*P*-value	*P*-value
Age (years)	<50	70.9	928		
	≥50	61.3	896	0.002*	NS
*BRCA1/2* mutation	Yes	66.9	315		
	No	66.3	1509	0.893	NS
Breast cancer risk based on genetic status and family history	Not increased	63.0	400		
	Increased	67.3	1424	0.191	NS
DCIS/breast cancer in history	Yes	62.2	662		
	No	68.6	1162	0.067	NS
Spontaneous nipple discharge	Yes	76.3	342		
	No	64.3	1482	0.001*	<0.001^§^
Breast size	A-B	72.8	394		
	≥C	64.6	1419	0.015*	0.039^§^
Parity	Nulliparous	66.5	497		
	Parous	66.4	1327	0.980	NS
Previous breast feeding	Yes	67.1	1056		
	No	65.4	768	0.616	NS
Duration of lactation (months)	≤6	68.0	530		
	>6	65.6	528	0.582	NS
Menstrual cycle day	1–14	72.9	322		
	≥14	73.9	365	0.780	NS
Age at menarche (years)	≤13	65.5	1158		
	>13	67.7	663	0.505	NS
Postmenopausal	Yes	61.0	1004		
	No	72.7	812	<0.001*	<0.001^§^
Current oral contraceptive use	Yes	75.3	247		
	No	65.1	1577	0.028*	NS
History oral contraceptive use	Yes	66.5	1652		
	No	64.1	168	0.671	NS
Intrauterine device	Yes	68.0	88		
	No	66.3	1728	0.801	NS
Current hormonal replacement therapy	Yes	77.8	42		
	No	66.1	1779	0.131	NS
History hormonal replacement therapy	Yes	75.8	129		
	No	65.6	1687	0.053	0.005^§^
Previous chemotherapy	Yes	63.4	293		
	No	67.0	1531	0.398	NS
Previous radiotherapy locally	Yes	61.0	257		
	No	67.2	1557	0.171	NS
Current anti-hormonal therapy	Yes	64.6	148		
	No	66.6	1667	0.678	NS
Previous anti-hormonal therapy	Yes	67.7	237		
	No	66.3	1586	0.746	0.014^§^
Previous breast surgery¶	Yes	61.6	415		
	No	67.7	1409	0.114	NS
Bilateral oophorectomy	Yes	64.9	355		
	No	67.9	1468	0.713	0.038^§^

^¶^ Including excision, breast conserving surgery, breast augmentation, breast reducing surgery

* Statistically significant predictor in univariate analysis

^§^ Statistically significant predictor in stepwise-backward multivariable analysis

In the 43 participants from whom nipple fluid was obtained with an electric breast pump, the procedure was successful in 62.3%. The success of obtaining fluid did not significantly differ between the manual or electric procedure in the same participant (*P* = 0.115), and the procedure takes on average 15 minutes less than manual aspiration.

### Discomfort and side effects of nipple fluid aspiration

**Fig 2:**
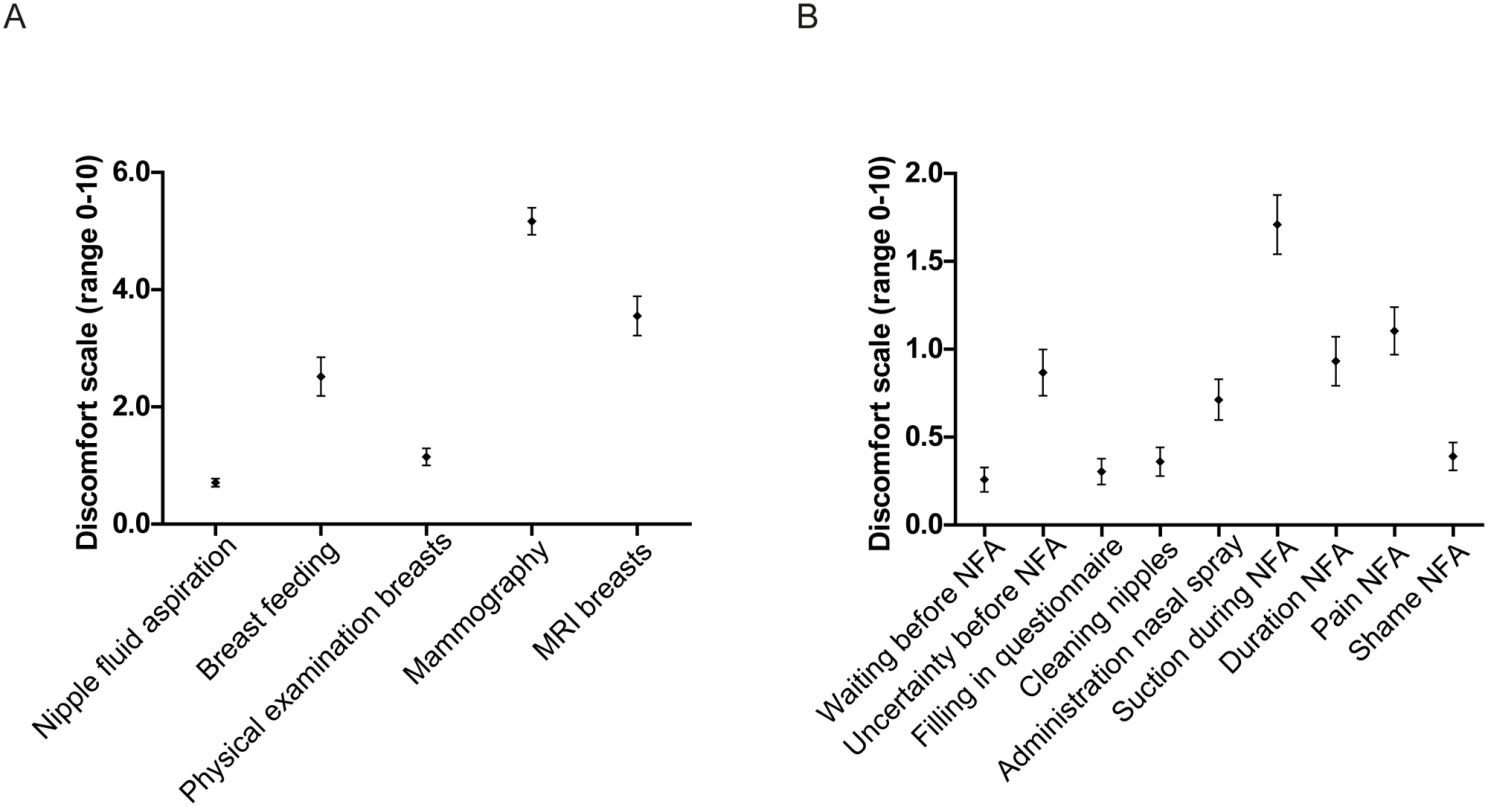
A, Discomfort of NFA compared to other breast cancer screening procedures (mean with 95% confidence intervals). B, Discomfort of different procedures during NFA (mean with 95% confidence intervals).

Fig 2A shows the discomfort scores of NFA and other procedures related to breast cancer care. The total discomfort of the entire NFA procedure in 451 aspirations during the first visit was on average rated at 0.71 (CI95% 0.64–0.78). This was significantly lower (all *P*-values <0.001; see also Fig 2A) than discomfort experienced during breast feeding (mean 2.51), physical breast examination (mean 1.15), mammography (mean 5.17), and MRI of the breasts (mean 3.55). Discomfort of the electric NFA was rated at 0.63, which did not significantly differ from the manual procedure in paired analyses of women undergoing both manual and electric NFA (*P* = 0.312). In Fig 2B discomfort scores of the different parts of the NFA procedure are shown. The application of vacuum was the most uncomfortable part of the procedure.

In Table 3 all reported side effects are listed. In 2.2% of the NFA procedures an adverse event was reported being potentially related to the procedure. All adverse events were mild and self-limiting. 99.5% of participants confirmed to be willing to repeat NFA and 97.3% would recommend the procedure to other women.

**Table 3:** Adverse events reported during and after 994 NFA procedures.

Adverse event	*N*	%
*Side effects probably related to NAF procedure*		
Sensitive breasts	7	0.7
Local irritation nipple or surrounding skin	4	0.4
Spontaneous nipple discharge after NFA	1	0.1
Cramps in uterus / abdominal discomfort	5	0.5
**Total**	**17**	**1.7**
*Side effects possibly related to NAF procedure*		
Nausea	2	0.2
Headache	1	0.1
Insomnia night after NFA procedure	2	0.2
**Total**	**5**	**0.5**

### Follow-up of participants

During follow-up, 141 women (31.3%) left the study (Table 4), either because women declined further participation (true drop-out) or follow-up ended if the follow-up period of 5 years was completed (5.5%), genetic testing did not show a *BRCA1/2* mutation (0.7%), women underwent preventive mastectomy (2.7%), or breast cancer developed. Twelve women developed breast cancer during follow up (2.7%) and three women died from disseminated breast cancer that developed during follow-up (0.7%). True drop-out was 20.0% and mainly caused by repeated unsuccessful aspiration attempts (10.9%), or adverse events (1.6%).

**Table 4:** Reasons for leaving the study of repeated NFA in 451 women at increased breast cancer risk.

Reason	*N*	%
*End of study*		
Completed follow-up period of 5 years	25	5.5
Breast cancer diagnosis during follow up^¶^	11	2.4
Preventive bilateral mastectomy during follow-up	11	2.4
No *BRCA1/2* mutation identified at genetic testing	3	0.7
Reductive mammoplasty including nipple reduction during follow-up	1	0.2
**Total**	**51**	**11.3**
*Drop-out*		
Repeated unsuccessful aspiration	49	10.9
Adverse events due to NFA procedure	7	1.6
- Vaginal candida*	1	
- Pain around nipple	4	
- Gorges around nipple	1	
- Sensitive breasts	1	
Too high burden of nipple fluid procedure because of general health	7	1.6
Lack of time	4	0.9
Afraid of recurrent nipple discharge	1	0.2
Other reason	22	4.9
**Total**	**90**	**20.0**

^¶^ One of the women that developed breast cancer during follow-up continued participating in the study.

* Developing vaginal candida after NFA is unlikely to be related to the procedure, however it was the reason to end the study for this participant

## Discussion

In this multicenter, prospective study, we annually collected nipple fluid for up to 5 consecutive years from women at increased breast cancer risk adhering to a surveillance program. We were able to obtain nipple fluid by vacuum aspiration and using oxytocin nasal spray in 66.4% of aspirated breasts. Annually repeated aspiration was feasible and very well tolerated with the occurrence of very few and self-limiting adverse events. Success rates were higher in premenopausal women, in women with a history of spontaneous nipple discharge, bilateral oophorectomy, previous hormonal replacement therapy or anti-hormonal therapy, and in women with smaller breast size. True drop-out was mainly due to repetitively unsuccessful aspiration.

Our first experience with NFA was obtained in healthy volunteers, where aspiration was successful in 94% of patients and in 84% per breast. Having spontaneous nipple discharge showed to be the only predictive factor in successfully obtaining nipple fluid. The procedure was well tolerated and no side effects from using oxytocin nasal spray were reported [20]. After this feasibility study, we started the prospective collection of nipple fluid in women at increased risk of breast cancer, our target population for introducing a potential new screening method. Preliminary results showed that NFA was also possible in this group of women [21]. In the present follow-up study, we show for the first time in a large group of high-risk women that repetitive NFA is a feasible and safe method. We could obtain nipple fluid in a clearly higher percentage compared to earlier studies in high risk women [26–28]. However, success rates were lower than observed in our earlier feasibility study in healthy volunteers [20]. The group of healthy volunteers consisted of younger women with a mean age of 29 years and only 12% of included women were postmenopausal, compared to 53% postmenopausal women in the present study. This difference may explain the higher success rates in our previous group of healthy volunteers.

In order to optimize adherence to nipple fluid aspiration as a new screening tool, we tested the feasibility of an electric breast pump, allowing women to obtain nipple fluid at home, in a subgroup of women. As the success rate was comparable to manual aspiration, we are currently optimizing the procedure for at home use, which may facilitate using NFA in a wider screening setting.

Other publications on obtaining breast fluid in high-risk women focused mainly on ductal lavage. In these studies NFA is solely used to identify fluid yielding ducts which can be cannulated by ductal lavage. Higgins *et al*. reported that fluid yielding ducts could be identified in 36% of high risk women (*N* = 33), in contrast to in 84% of women without an increased risk. Reduced yield of nipple fluid was associated with postmenopausal state, *BRCA* germline mutation and a history of risk reducing strategies such as PBSO or use of selective estrogen receptor modulator inhibitors. The authors hypothesized that endocrine mechanisms associated with risk-reducing therapies could explain the diminished production of nipple fluid [26]. Mitchell *et al*. identified fluid yielding ducts in 60% of *BRCA* mutation carriers (*N* = 52) and again postmenopausal status was associated with less fluid yielding [27]. Twelves *et al*. also studied women at increased breast cancer risk (*N* = 67), but did not include women with a known *BRCA* mutation. Nipple fluid was produced in 83% in at least one duct. Following NFA, 77% of ducts were cannulated for ductal lavage of which 83% produced samples with adequate cellularity. In 40% women experienced mild discomfort after ductal lavage. One women developed mild breast inflammation, resolving after antibiotics. Total drop-out rate was 21%. Withdrawal occurred in 3 women because of intolerance of the procedure and in another 3 by anxiety and pain [28]. These studies show that breast fluid can be obtained in high risk women, but success rates vary. Moreover, discomfort of ductal lavage is considerably higher than we experienced after our non-invasive nipple fluid aspiration.

An important difference between the present and earlier studies is the use of oxytocin nasal spray, which may explain higher success rates. Oxytocin is a hormone which plays a key role in the contraction of the uterus during parturition. Moreover, oxytocin is important in the ejection of milk from the mammary gland during breast feeding. In the present study the incidence of reported side effects was low. In 1.7% side effects were probably related to the aspiration procedure and in 0.5% possibly. The adverse events were mild and self-limiting in all cases. Abdominal discomfort was reported in 0.5% of aspirations after the procedure, which could be specifically related to the use of oxytocin. Long-term effects were not observed, which is in accordance with the findings of the use of long-term oxytocin intranasally in male children with autism (8–24 IU/dose) [29]. In the study by Zhang *et al*., 9 healthy women were given one spray of oxytocin in both nostrils (total dose 50 IU) before NFA and no adverse events were reported [30]. This makes the use of oxytocin safe and helpful in obtaining nipple fluid.

A limitation of NFA applicability in screening programs is that, at this point, fluid can be obtained in 66% of the breasts aspirated. To increase nipple fluid yielding, it is important to get more insight into the determinants affecting successful aspiration. It has been shown that the yielding of nipple fluid is associated with higher prolactin, regardless of parity and menopausal status [31]. Together with our and other findings that NFA is more successful in premenopausal women, this implies that endocrine environment is important in nipple fluid yielding. Another important note is that we only performed one nipple fluid aspiration attempt per participation year. Studies describing multiple attempts in women with both standard and increased breast cancer risk, report a 94% or higher success rate in obtaining nipple fluid [32]. This implies that multiple aspirations might increase successful nipple aspirations and this further increases the necessity for self-testing at home.

Nipple fluid contains cells and free DNA, which makes it suitable for the detection of different biomarkers such as methylation [33–37], proteins [38], and hormones [39]. Although low nipple volumes may hamper a multidisciplinary biomarker approach, we believe much of its limitations can be overcome due to the continuous development of increasingly sensitive techniques, and all-in-one DNA/RNA/protein isolation methods. Besides, we have already demonstrated that methylation analysis in nipple fluid samples from high risk women is feasible [21]. At this moment we are analysing the nipple fluid samples to investigate if the process of breast carcinogenesis can be predicted by the occurrence of methylation aberrations. Moreover, we collected nipple fluid samples from healthy volunteers and breast cancer patients for comparison. Results from nipple fluid analyses will be reported in a separate paper.

In conclusion, sampling of nipple fluid as breast-derived material with oxytocin-assisted aspiration is a feasible and promising approach yielding a valuable source of breast cancer specific biomarkers. Since sampling of nipple fluid is feasible in a screening population, the samples could be used for many different purposes like methylation, protein, or hormone biomarkers analysis.
